# The incidence of total hip arthroplasty after hip arthroscopy in osteoarthritic patients

**DOI:** 10.1186/1758-2555-2-18

**Published:** 2010-07-29

**Authors:** Barak Haviv, John O'Donnell

**Affiliations:** 1 Department of Orthopaedic Surgery, Arthroscopy and Sports Injuries Unit, Rabin Medical Center, 39 Jabotinski Street, Petach-Tikva 49100, Israel; 2 Department of Orthopaedic Surgery, St Vincent and Mercy Private Hospital, 159 Grey Street, East Melbourne 3002, Victoria, Australia

## Abstract

**Objective:**

To assess the incidence of total hip arthroplasty (THA) in osteoarthritic patients who were treated by arthroscopic debridement and to evaluate factors that might influence the time interval from the first hip arthroscopy to THA.

**Design:**

Retrospective clinical series

**Methods:**

Follow-up data and surgical reports were retrieved from 564 records of osteoarthritic patients that have had hip arthroscopy between the years 2002 to 2009 with a mean follow-up time of 3.2 years (range, 1-6.4 years). The time interval between the first hip arthroscopy to THA was modelled as a function of patient age; level of cartilage damage; procedures performed and repeated arthroscopies with the use of multivariate regression analysis.

**Results:**

Ninety (16%) of all participants eventually required THA. The awaiting time from the first arthroscopy to a hip replacement was found to be longer in patients younger than 55 years and in a milder osteoarthritic stage. Patients that experienced repeated hip scopes had a longer time to THA than those with only a single procedure. Procedures performed concomitant with debridement and lavage did not affect the time interval to THA.

**Conclusions:**

In our series of arthroscopic treatment of hip osteoarthritis, 16% required THA over a period of 7 years. Factors that influence the time to arthroplasty were age, degree of osteoarthritis and recurrent procedures.

## Background

Currently there are various options to treat osteoarthritis (OA) and certain evidence based recommendations have been developed [1,2]. According to these propositions in young adults with symptomatic OA one should consider a joint preserving surgical procedure while replacement is usually reserved for older patients. With the evolution of hip arthroscopy, it has been used as joint preserving surgery for OA among various other indications, yet there are only a few reports on its efficacy in treating OA [3-8]. In a controlled trial involving patients with osteoarthritis of the knee [9], the outcomes after arthroscopic lavage or arthroscopic debridement were no better than those after a placebo procedure. There are, however, times when arthroscopic treatment of the osteoarthritic joint can be of benefit, particularly in that patient who has relatively mild to moderate osteoarthritis and a mechanically significant derangement [10]. Several studies do support the use of hip arthroscopy in mild to moderate OA while others consider severe OA as a contraindication for hip arthroscopy [11,12]. This study reviews a cohort of patients that required a total hip arthroplasty (THA) after a trial of arthroscopic surgery for hip degeneration. The aim of the study was to assess the incidence of THA in that subgroup of patients and to evaluate several factors that might influence the time interval from the first hip arthroscopy to THA. Our hypothesis is that in selected patients hip arthroscopy can temporarily delay the need for replacement.

## Methods

The inclusion criteria for the study were patients who have had hip replacement following a trial of arthroscopic surgery for idiopathic osteoarthritis (i.e. not secondary to infection, trauma or avascular necrosis). The indication for the first hip arthroscopy was hip pain with limitation of internal rotation and confirmative findings on radiography (i.e. Tönnis grade 1 to 3), not responsive to a non operative treatment for at least 12 weeks. From our database of 2628 hip arthroscopies since 2002, 564 cases were treated for osteoarthritis. Of those, 90 subsequently required total hip arthroplasty (THA). We retrospectively reviewed the files and operation reports of patients that had hip arthroscopic surgery done for OA in the years 2002 to 2007 with a mean follow-up time of 3.2 years (range, 1-6.4 years). The information that was retrieved included demographic details and arthroscopic findings that were observed and treated. All patients were informed that their charts and images might be reviewed for scientific purposes and given the opportunity to forbid such use of their data. All patients included in our study consented to the use of their data. Repeated arthroscopy was considered if the patient suggested it himself because of recurrent hip pain and there was no radiographic evidence of significant deterioration. Hip replacement post arthroscopy was considered if the symptoms had worsened for at least 12 weeks, in spite of appropriate conservative treatment.

All arthroscopies were done by a single surgeon well experienced in that procedure. We used the lateral decubitus position, general anesthesia (without muscle relaxants) and traction for the operated hip in a technique described by Mason et al [13]. Arthritic hips were treated by chondroplasty, removal of loose bodies, synovectomy and Ligamentum Teres debridement. Occasionally concomitant procedures were performed as necessary (Table 1). Femoral osteectomy was done for osteophyte or impingement lesion correction. Any localized acetabular lesion of less than 3 to 4 cm^2 ^was treated by microfracture [14]. At the end of the surgical procedure, the joint was lavaged and injected with local anesthetic (Bupivacaine 100 mg in earlier cases, and Ropivacaine 150 mg in later ones) and Morphine 5 mg. Betamethasone 11.4 mg was also injected if there had not been any bone resection. Postoperatively, weight bearing as tolerated was advised on the surgically treated limb, with crutches for the first few days as required. Follow up was done at 1 week and 6 weeks after surgery for all patients. After this period, additional appointments were made with patients for whom it was deemed necessary.

**Table 1:** Percentage of concomitant procedures in addition to arthroscopic debridement and lavage

Procedures	% of THA group	% of non-THA group
**Femoral osteectomy**	16	31
**Labral repair**	7	7
**Microfracture**^**a**^	11	5
**Debridement and Lavage**^**b**^	76	67

^a^Since 2007

^b^Treated by chondroplasty, removal of loose bodies, synovectomy and debridement of Ligamentum Teres.

Abbreviations: THA, total hip arthroplasy

**Figure 1:**
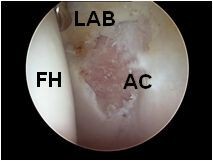
**Arthroscopic osteoarthritis**. The acetabular rim surface is involved (Abbreviations: FH, femoral head; AC, acetabulum; LAB, labrum).

There is, as yet, no generally accepted arthroscopic staging system for osteoarthritis. We defined the degrees of arthroscopic OA according to the damaged surface area (Figure 1): mild when less than 30% of the acetabular anterior wall width was involved with full thickness articular cartilage loss (i.e. Outerbridge [15] Grade 3, 4); moderate if it was more than 30% and severe if the femoral head was involved as well.

The time interval between the first hip arthroscopy to THA was modelled as a function of patient age; level of cartilage damage; procedures performed and repeated arthroscopies with the use of multivariate regression analysis. The impact of the above independent variables on the survival time (i.e. until THA) was also verified by a multivariate Cox proportional hazards model. Correlation between time intervals and the different variables was investigated by Pearson's correlation coefficient test. We considered p to be statistically significant if it was less than 0.05.

## Results

### Incidence of Total Hip Arthroplasties

**Figure 2:**
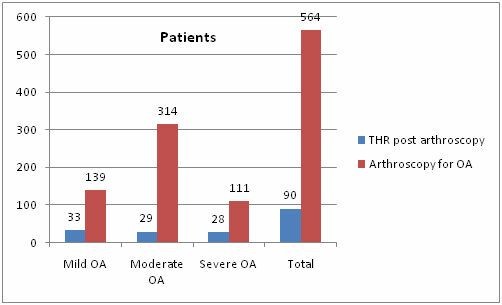
**A graph shows the number of patients in each of the osteoarthritis categories and in total (Abbreviations: OA, osteoarthritis; THR, total hip replacement)**.

Overall, 564 hip arthroscopies done for OA were included in the study (Figure 2). We found that 90 of them (16%) deteriorated to hip replacements between the years of 2002 to 2009. The survival probability (SP) to avoid THA was calculated with the Cox regression model. At one year after the index arthroscopic operation the SP was 94% (p = 0.002), at 3 years was 88% (p = 0.005) and at 6 years was 84% (p = 0.007). There were 35 males and 55 females. The mean age was 55 (range, 32-80). It is noteworthy that 75% of the patients with severe OA did not require THA by the end of the study.

### Time Interval to THA

The mean awaiting time from the first arthroscopy to THA was 1.5 years (range, 0.06-5.1 years). Several factors were found to affect this time interval by utilizing multivariate regression analysis (Table 2). The time interval from scope to replacement had a reverse correlation to the level of arthritis (Pearson's correlation coefficient r = -0.35, 95% CI = -0.52 to -0.16, p < 0.001). The mean time interval for mild OA was 2.2 years (range, 0.2-5 years). The mean time interval for moderate OA was 1.2 years (range, 0.2-4.4 years). The mean time interval for severe OA was 1.1 years (range, 0.1-5.1 years), P value < 0.05.

**Table 2:** Correlation between variables and the time interval to total hip arthroplasty in patients that were treated by hip arthroscopy

**Pearson correlations**^**a**^		
**Variable**	**Time to THA**	**P value**

Age	-0.34	0.013
Level of cartilage damage	-0.35	0.011
Multiple scopes	0.47	< 0.001
Procedures	-0.1	0.35

^a^Multivariable analysis using a linear regression model with backwards selection and the time between arthroscopy to THR as the dependent variable → R^2 ^= 0.32, R = 0.57

Abbreviations: THA, total hip arthroplasty

There was a reverse correlation between age and the time difference between the two surgeries (r = -0.34, 95%CI = -0.51 to -0.14, p = 0.001). We have tested two different age groups according to the median age of the study population (i.e. 55 year old). There were 45 patients in the under 55 years group and 45 in the over 55. The time interval from the first arthroscopy to a hip replacement was longer in patients younger than 55 years with a mean time of 1.9 years (range, 0.2-5.1 years) than those who were older with a mean time of 1.2 years (range, 0.1-3.5 years), P value = 0.004.

From the subgroup of patients that eventually required THA 16% have had femoral osteoplasty concomitant to their first arthroscopic debridement in comparison to 31% of patients who did not require THA. Conversely, concomitant procedures did not correlate with the awaiting time to THA.

Ten of the 90 patients have had repeated arthroscopies (2-4) with a mean time of 2.6 years (range, 0.8-4.7 years) to hip replacement compared to a mean of 1.3 years (range, 0.1-5.1 years) in those who had a single procedure, P value < 0.05. The mean age of this group of patients was 52 years (range, 32-76 years). Five of them had moderate to severe osteoarthritis at the first arthroscopy.

## Discussion

This study shows a relatively low incidence of total hip arthroplasy (THA) in patients that were treated by arthroscopic debridement and lavage for osteoarthritis. Byrd and Jones in their prospective long term follow-up reported on 14 (27%) out of 52 cases of hip arthroscopies who were converted to THA [16]. Philippon et al have had 10 (9%) conversions to THA out 112 after a mean follow-up time of 16 months [17]. The use of arthroscopic debridement for the treatment of arthritis remains controversial, and its efficacy has not been demonstrated by high-quality trials.

The limiting factor in treatment outcome in many mechanically compromised hips is the amount of cartilage damage that has occurred before the surgery [18,19]. Unfortunately early degenerative changes which are revealed at arthroscopy are often not apparent on a regular radiograph [7] and although both MRI and CT scanning are more sensitive, the diagnosis may be basically clinical. Hip arthroscopy can serve as a diagnostic and therapeutic tool in these cases. In our study we excluded patients with no radiographic signs of OA (i.e. Tönnis 0). That might explain the relatively high percentage of patients (23%) with mild (less than 30% of acetabular anterior wall involvement) that needed a hip replacement, since many small or partial thickness cartilaginous lesions who were omitted from the study settle following debridement and microfracture [20] and others may represent a mild pre arthritic condition.

One of the obstacles in conducting a comparative study on arthroscopic hip degeneration findings is an accurate and reproducible staging system. Many articles quote the Outerbridge grading classification of chondral lesions [15] which is not specific for OA and the Tönnis Classification [21] that represents radiographic changes. Based on our experience with arthroscopic treatment for hip arthritis we defined three levels of chondral damage according to the proportion of joint involvement. The aim of our study was to assess the factors that may influence the awaiting time to arthroplasty after arthroscopic treatment for osteoarthritis in its different stages. This study follows the course of 564 osteoarthritic patients that were treated by arthroscopic surgery over a time period of 7 years and scrutinizes 90 (16%) of them that had their hip replaced. Surgery included synovectomy, debridement and occasionally removal of femoro-acetabular-impingement (FAI) lesions. Addressing FAI lesions was shown to be valuable in patients with early OA by Kim et al [22]. They concluded that arthroscopic treatment of osteoarthritis of the hip fails if there is detectable femoro-acetabular impingement. In our study concomitant procedures did not influence the time interval from the first hip arthroscopy to THA; however, patients that did not deteriorate to THA had higher percentage of concomitant femoral osteoplasties performed.

The time interval was found to be conversely related to the level of arthritis noted in the first arthroscopy. The mean time interval for mild OA was 2.2 years (range, 0.2-5 years) whereas for severe OA it was 1.1 years (range, 0.1-5.1 years). These findings correlate with previous publications on short term results [3-6]. In addition, Margheritini and Villar [5] showed that young patients with early OA were those associated with a higher rate of procedure success, similar to our observation of longer time intervals to THA in patients younger than 55.

Ten of the 90 individuals who required THA underwent prior repeated arthroscopies. This finding suggests that these individuals were sufficiently satisfied with the first operation to have the procedure again. Although this can represent a selection bias towards younger patients with milder degenerative changes, the mean age and degree of osteoarthritis in these individuals were not much different from the others in our study. In contrast, Helenius et al [3] have shown that hip arthroscopy for osteoarthritis can provide a temporary relief but repeated arthroscopies had no therapeutic effect.

To our knowledge this study represents the largest series of hip arthroscopies on osteoarthritis published so far; however, important clinical variables such as patient satisfaction, risk perception, and functional outcome were not available in the database that we used. Another limitation is the fact that all procedures were performed by the same surgeon which might not reflect other people's results. Future investigators should consider performing clinical evaluation by means of comparative scores to test the clinical outcome of osteoarthritic patients treated by arthroscopic debridement.

## Conclusions

In our series of hip arthroscopies performed for osteoarthritis, there was a 16% chance of progressing to a hip replacement. There was a longer time interval between arthroscopy and total hip arthroplasty in those individuals with younger age and milder arthritis. Repeated arthroscopy may provide some clinical short-term benefit.

## Competing interests

The authors declare that they have no competing interests.

## Authors' contributions

JO participated in the design and co-ordination of the study and conducted the clinical evaluations of all patients in addition to undertaking the arthroscopic surgery in the entire population. BH participated in the design and co-ordination of the study, retrieved the required data from the database and undertook the statistical analysis. Both authors participated in drafting and revising of the manuscript and also read and approved the final manuscript.
